# A Multi-Constraint Co-Optimization LQG Frequency Steering Method for LEO Satellite Oscillators

**DOI:** 10.3390/s25154733

**Published:** 2025-07-31

**Authors:** Dongdong Wang, Wenhe Liao, Bin Liu, Qianghua Yu

**Affiliations:** 1 School of Mechanical Engineering, Nanjing University of Science and Technology, Nanjing 210094, China; 2 Beijing Research Institute of Telemetry, China Aerospace Science and Technology Corporation, Beijing 100094, China; binaryliu@126.com (B.L.); yuqh@brit.com.cn (Q.Y.)

**Keywords:** low Earth orbit (LEO) satellites, oven-controlled crystal oscillators (OCXOs), multi-constraint co-optimization, LQG control, frequency stability

## Abstract

High-precision time–frequency systems are essential for low Earth orbit (LEO) navigation satellites to achieve real-time (RT) centimeter-level positioning services. However, subject to stringent size, power, and cost constraints, LEO satellites are typically equipped with oven-controlled crystal oscillators (OCXOs) as the system clock. The inherent long-term stability of OCXOs leads to rapid clock error accumulation, severely degrading positioning accuracy. To simultaneously balance multi-dimensional requirements such as clock bias accuracy, and frequency stability and phase continuity, this study proposes a linear quadratic Gaussian (LQG) frequency precision steering method that integrates a four-dimensional constraint integrated (FDCI) model and hierarchical weight optimization. An improved system error model is refined to quantify the covariance components (Σ_11_, Σ_22_) of the LQG closed-loop control system. Then, based on the FDCI model that explicitly incorporates quantization noise, frequency adjustment, frequency stability, and clock bias variance, a priority-driven collaborative optimization mechanism systematically determines the weight matrices, ensuring a robust tradeoff among multiple performance criteria. Experiments on OCXO payload products, with micro-step actuation, demonstrate that the proposed method reduces the clock error RMS to 0.14 ns and achieves multi-timescale stability enhancement. The short-to-long-term frequency stability reaches 9.38 × 10^−13^ at 100 s, and long-term frequency stability is 4.22 × 10^−14^ at 10,000 s, representing three orders of magnitude enhancement over a free-running OCXO. Compared to conventional PID control (clock bias RMS 0.38 ns) and pure Kalman filtering (stability 6.1 × 10^−13^ at 10,000 s), the proposed method reduces clock bias by 37% and improves stability by 93%. The impact of quantization noise on short-term stability (1–40 s) is contained within 13%. The principal novelty arises from the systematic integration of theoretical constraints and performance optimization within a unified framework. This approach comprehensively enhances the time–frequency performance of OCXOs, providing a low-cost, high-precision timing–frequency reference solution for LEO satellites.

**Dataset:** DOI number or link to the deposited dataset in cases where the dataset is published or set to be published separately. If the dataset is submitted and will be published as a supplement to this paper in the journal *Sensors*, this field will be filled by the editors of the journal. In this case, please make sure to submit the dataset as a supplement when entering your manuscript into our manuscript editorial system.

**Dataset License:** license under which the dataset is made available (CC0, CC-BY, CC-BY-SA, CC-BY-NC, etc.).

## 1. Introduction

Low Earth orbit (LEO) satellites, characterized by low orbital altitude, rapid geometry variations, and strong Earth-received signal strength, constitute a critical technology for enhancing Global Navigation Satellite System (GNSS) service accuracy to enable real-time centimeter-level positioning services [1]. However, to achieve this ultra-precise positioning capability, stringent demands are imposed on satellite timing references. Oven-controlled crystal oscillators (OCXOs) are typically selected as the time–frequency reference for LEO satellites due to constraints in mass, cost, and power consumption [2]. While OCXOs exhibit excellent short-term frequency stability, the long-term stability is susceptible to inherent aging drift, random noise, and environmental disturbances. This inherent limitation makes it challenging to maintain constant frequency over extended durations, leading to rapid accumulation of clock bias. For LEO navigation, 1 ns clock error directly induces 0.3 m ranging error (range = c × t, c = 2.99792458 × 10^8^ m/s). Consequently, positioning, navigation, and timing (PNT) service accuracy is severely constrained by clock error level. The implementation of a robust real-time control system for the OCXO-based time–frequency reference is essential to meet the high-accuracy, high-stability requirements for rapid precision positioning services.

The pursuit of high-precision OCXO control under stringent conditions for real-time precision positioning presents multi-dimensional challenges. Frequency control must simultaneously address rapid response, process noise suppression, short-term stability maintenance, and long-term drift mitigation. Overly aggressive strategies, while enabling fast frequency deviation correction, introduce short-term phase perturbations. Conversely, conservative approaches struggle to suppress long-term drift and fail to concurrently satisfy stability requirements across multiple timescales.

Extensive research has been conducted globally to address these challenges in satellite clock control. Proportional integral derivative (PID) control-based steering loops are commonly employed for basic phase synchronization through feedback regulation [3,4]. Fixed-offset frequency correction [5] or second-order servo loops [6] are typically utilized in these implementations. However, these often fail to deliver optimal performance in complex noisy environments due to predominant reliance on empirical parameter tuning [7]. Critically, PID-based methods cannot simultaneously satisfy multi-objective requirements such as short-term stability (STS) and long-term stability (LTS).

Alternative statistical estimation approaches, including Kalman filtering [8,9,10,11,12,13] and least-squares fitting [14], periodically inject correction values to adjust the clock through prediction-update mechanisms. While frequency stability characteristics are improved, clock bias (RMS) typically remains at the nanosecond level due to inherent estimation delays. Multi-source data fusion strategies effectively enhance clock bias accuracy by integrating multi-GNSS observations [15], inter-satellite links [16], and ground station references [17]. Although effective for clock noise suppression, these approaches are constrained by slow-update corrective mechanisms with refresh rates exceeding 60 s, which are primarily oriented toward enhancing phase accuracy in pulse-per-second (PPS) signal alignment rather than comprehensive frequency stability control.

Recent studies introduce the optimal control theory into time–frequency synchronization [18], such as LQG control for atomic clock calibration [19] and unbiased FIR filtering for robustness [20,21]. Some studies have further evolved LQG-based clock control methods using GNSS timing observations [22]. Nevertheless, existing approaches predominantly focus on single-metric optimization (e.g., clock bias error or stability), leading to inherent tradeoffs between control precision and stability. Furthermore, the steering method often depends on empirical parameters with limited adaptability to real-time OCXO adjustments required for centimeter-level positioning. In addition, a systematic theoretical framework addressing multi-dimensional performance-constrained control of high-stability oscillators in fast precision positioning applications remains unaddressed. Therefore, a fundamental multi-constraint control framework capable of simultaneously optimizing clock bias, short-term stability, and long-term drift is imperative.

To resolve these issues, this study proposes a LQG frequency precision-steering method based on multi-parameter constraints and weight matrices co-optimization. A LQG control error model incorporating quantization noise introduced during frequency adjustments and intrinsic oscillator noise is established. Analytical expressions for time–frequency metrics for precision positioning services and the covariance of the closed-loop control system are derived. The accuracy requirements in the time–frequency domain are transformed into constraint sets for control optimization. Hierarchical optimization of weight matrix parameters under multiple constraints ultimately yields globally optimal solutions, enhancing OCXO stability under real-time positioning demands. Experimental results demonstrate the significant improvements in clock bias accuracy and frequency stability for the OCXO time–frequency systems. It meets the stringent requirements for real-time centimeter-level positioning services, providing a systematic solution for OCXO clock management in LEO satellites.

## 2. System Basic Principle

**Figure 1:**
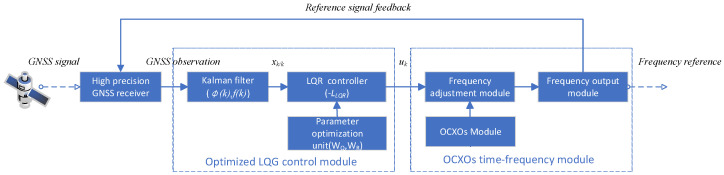
The optimized LQG frequency control system model.

The proposed LQG frequency control system model integrates multi-constraint optimization with real-time feedback, as illustrated in Figure 1. The system comprises three core modules: (1) a state estimation unit where a Kalman filter is employed to process GNSS observations and predict clock errors; (2) a parameter optimization unit in which the FDCI constraint hierarchy (Section 3.3) is implemented; and (3) an actuation module where micro-step frequency adjustments are executed.

Following this model structure, the OCXO model (Section 2.1) is established to characterize its dynamic behavior, which captures frequency stability via clock bias $\varphi \left(k\right)$ and frequency deviation $f\left(k\right)$. Subsequently, the LQG controller (Section 2.2), which encompasses Kalman filtering estimation and LQR regulation to minimize process noise, is elaborated to form the theoretical foundation for the multi-constraint optimization framework introduced in Section 3. Especially within the parameter optimization unit, as described in detail in Section 3.4, weight adjustment is executed to balance between quantization noise limits (D1), phase continuity (*D*2), stability bounds (*D*3), and accuracy requirements (*D*4). Optimized frequency commands are subsequently dispatched to the frequency adjustment module for micro-step actuation, while real-time performance feedback is routed back to enable adaptive tuning. Finally, the corrected frequency signals are output by the OCXO’s time–frequency module as a stable reference. Real-time adaptability to LEO orbital dynamics is ensured through this integrated framework.

### 2.1. System Clock State Modeling

The dynamic behavior of oven-controlled crystal oscillators (OCXOs) is characterized by a two-state variable model [23,24] composed of clock bias *φ*(*k*) and frequency deviation *f*(*k*). The state vector $x_{k}$ is described as follows:

(1) $$x_{k}={\left\lceil \varphi \left(k\right),\;f\left(k\right)\right\rceil }^{T},$$

Under closed-loop control, the discrete time state–space model is described as follows:

(2) $$x_{k+1}={Ax}_{k}+{Bu}_{k}+\omega_{k},$$

The system matrices are given by:

(3) $$A=\left[\begin{array}{cc} 1 & \tau \\ 0 & 1 \end{array}\right],\;B=\left[\begin{array}{c} 0\\ 1 \end{array}\right]$$

where τ is the sampling interval (typically 1 s in LEO systems). The sampling interval τ is typically set to 1 s to align with LEO orbital dynamics, ensuring that state updates synchronize with GNSS observation cycles (1 s). This configuration achieves centimeter-level positioning accuracy in dynamic environments [25]. With a 1 Hz update, the clock-estimation filter corrects errors every second, preventing accumulation of unmodeled drift. Frequent feedback stabilizes the solution by tightly bounding clock bias estimates. If observations are too infrequent (e.g., 30 s or 300 s), the clock can drift between updates, leading to larger corrections that may reduce convergence accuracy. In contrast, a 1 s sampling interval yields a smoother, continuous tracking of the clock bias, improving both estimation accuracy and solution stability.

The process noise $\omega_{k}$~*N* (0, *Q*) is a zero-mean Gaussian process composed of power-law noise types such as white noise, flicker noise, and random walk noise.

Consequently, the state vector $x_{k}$ encapsulates clock bias ($\varphi \left(k\right)$) and frequency deviation ($f\left(k\right)$), with matrix *A* modeling the sampling interval *τ* = 1 s (selected for compatibility with LEO GNSS observations, as detailed in Section 4.1).

The power spectral density of $\omega_{k}$ is modeled by a combination of power-law components [26]:

(4) $$S_{y}\left(f\right)=\left\{\begin{array}{c} \sum_{\alpha =-2}^{2}h_{\alpha }f^{\alpha },0<f<f_{h}\\ 0,\;\;\;\;\;\;\;\;\;\;\;\;f>f_{h} \end{array}\right.,$$

where $S_{y}\left(f\right)$ is power spectral density, $f_{h}$ is the high-frequency cutoff, $h_{\alpha }$ is noise intensity coefficients, $h_{-2}$ is random walk frequency modulation (RWFM), $h_{-1}$ is flicker frequency modulation (FFM), $h_{0}$ is white frequency modulation (WFM), $h_{1}$ is flicker phase modulation (FPM), and $h_{2}$ is white phase modulation (WPM).

For each noise type, the integral yields a closed-form expression, and the total Allan variance is [27]:

(5) $$\sigma_{y}^{2}\left(\tau \right)=\frac{2\pi^{2}\tau }{3}h_{-2}+2\ln\left(2\right)h_{-1}+\frac{h_{0}}{2\tau }+\frac{3\left[\gamma +\ln\left(2\pi f_{H}\tau \right)\right]-\ln\left(2\right)}{4\pi^{2}\tau^{2}}h_{1}+\frac{3f_{H}}{4\pi^{2}\tau^{2}}h_{2}$$

where γ ≈ 0.5772 is the Euler–Mascheroni constant and $f_{H}$ is the high-frequency cutoff with $f_{H}\tau \gg 1/2\pi$.

The process noise covariance matrix Q can be written as:

(6) $$Q=\left[\begin{array}{cc} \frac{h_{0}\tau^{3}}{3}+h_{1}\ln\left(4\right)+h_{2}\tau +\frac{h_{-2}\tau^{3}}{3} & \frac{{h_{0}\tau }^{2}}{2}+\frac{h_{-2}\tau^{4}}{8}\\ \frac{{h_{0}\tau }^{2}}{2}+\frac{h_{-2}\tau^{4}}{8} & h_{0}\tau +{4h}_{-1}\ln\left(2\right)+h_{-2}\tau \end{array}\right]$$

Equation (6) integrates all five noise types into a unified process noise model for OCXOs. The dominant noise type at each τ interval can be inferred from the slope of the log–log Allan deviation curve according to Equation (5) [28].

### 2.2. Linear Quadratic Gaussian Control

The structure of the LQG controller consists of a Kalman estimator, which provides the optimal state estimation under stochastic noise and an LQR controller, which applies optimal feedback to the estimated state [29]. It provides an effective solution for designing closed-loop controllers in the presence of noise and model uncertainties, particularly suitable for oscillator frequency stabilization.

#### 2.2.1. Kalman Filter

The Kalman filter recursively updates both the state estimate and its associated covariance, based on the prediction from the system model and correction using the latest measurement [30].

The observation equation is:

(7) $$y_{k}={Cx}_{k}+v_{k},$$

where $y_{k}$ is the measurement vector at time k and $C=\left[\begin{array}{cc} 1 & 0 \end{array}\right]$ is observation matrix. $v_{k}$∼N (0, R) is the measurement noise, assumed to follow a white Gaussian distribution with zero mean and covariance R. Additionally, the iterative state estimation involves five key steps [31], which follow:

Step 1: State Prediction ${\overset{´}{x}}_{k+1/k}$

(8) $${\overset{´}{x}}_{k+1/k}=A{\overset{´}{x}}_{k/k}+{Bu}_{k},$$

where ${\overset{´}{x}}_{k+1/k}$ is the predicted state at time k + 1, based on information up to time k.

Step 2: Covariance Prediction $P_{k+1/k}$

(9) $$P_{k+1/k}=AP_{k/k}A^{T}+Q,$$

where $P_{k+1/k}$ denotes the covariance of the predicted estimate, $P_{k/k}$ is the covariance of the previous estimate, and Q is the process noise covariance.

Step 3: Kalman Gain Computation $K_{kalman}$

(10) $$K_{kalman}=P_{k+1/k}C^{T}{\left(CP_{k+1/k}C^{T}+R\right)}^{-1},$$

The Kalman gain matrix $K_{kalman}$ dynamically adjusts how much the observation influences the final estimate.

Step 4: State Update (Measurement Correction) ${\overset{´}{x}}_{k+1/k+1}$:

(11) $${\overset{´}{x}}_{k+1/k+1}={\overset{´}{x}}_{k+1/k}+K_{kalman}(y_{k}-C{\overset{´}{x}}_{k+1/k}),$$

where $y_{k}$ is the actual measurement and $y_{k}-C{\overset{´}{x}}_{k+1/k}$ is the innovation.

Step 5: Covariance Update $P_{k+1/k+1}$

(12) $$P_{k+1/k+1}=(I-K_{kalman}C)P_{k+1/k},$$

where I is the identity matrix of appropriate dimension.

#### 2.2.2. Linear Quadratic Regulator (LQR)

A cost function is defined as [32]:

(13) $$J=E\left[\sum_{k=0}^{\infty }\left(x_{k}^{T}W_{Q}x_{k}+u_{k}^{T}W_{R}u_{k}\right)\right],$$

Here, $W_{Q}=\left[\begin{array}{cc} q_{11} & q_{12}\\ q_{21} & q_{22} \end{array}\right]$ is the positive semi-definite weighting matrix for state deviation, and $W_{R}=r$ is the positive definite weighting matrix for control energy.

The optimal gain matrix $L_{LQR}=\left[L1,\;\;L2\right]$ is computed as [33]:

(14) $$L_{LQR}={\left(B^{T}SB+W_{R}\right)}^{-1}B^{T}SA,$$

where S is the unique positive semi-definite solution to the discrete algebraic Riccati equation [34]:

(15) $$S=A^{T}SA-A^{T}SB{\left(B^{T}SB+W_{R}\right)}^{-1}B^{T}SA+W_{Q},$$

Since the true state $x_{k}$ is not directly available, the estimated state ${\overset{´}{x}}_{k/k}$ is used in the control. The control input $u_{k}$ is:

(16) $$u_{k}=-L_{LQR}{\overset{´}{x}}_{k/k},$$

## 3. The LQG Closed-Loop Control Method Based on Multi-Constraint Optimization

The clock-state equation under the LQG control is given by:

(17) $$\;\;x_{k+1}=({A-BL_{LQR})x}_{k}+\omega_{k},$$

where $x_{k}$ and $x_{k+1}$ are the system state vectors at time k and k + 1, $A-BL_{LQR}$ is the closed-loop state transition matrix, and $\omega_{k}$ is the process noise

### 3.1. Quantization Noise Analysis in Frequency Precision Steering

Due to digital quantization in practice actuation, the true control input $u_{k}^{*}$ is an integer multiple of the minimum adjustment $u_{min}$. Therefore, $u_{k}^{*}$ is calculated as:

(18) $$u_{k}^{*}=u_{min}\cdot round\left(\frac{u_{k}}{u_{min}}\right),$$

The adjustment precision is $u_{min}$/2, and the quantization error $\varepsilon_{k}$ is defined as:

(19) $$\varepsilon_{k}=u_{k}^{*}{-u}_{k}=u_{min}\cdot round\left(\frac{u_{k}}{u_{min}}\right)-u_{k},$$

where $\varepsilon_{k}$ manifests as quantization noise in the closed-loop system, following a uniform distribution $\varepsilon_{k}\sim u(-\frac{u_{min}}{2},+\frac{u_{min}}{2})$, with zero mean [35].

The corresponding quantization noise covariance $\Sigma_{\varepsilon }$ is:

(20) $$\Sigma_{\varepsilon }=\frac{{u_{min}}^{2}}{12},$$

### 3.2. Multi-Source Error Propagation Modeling

The state estimation error $e_{k}$ is defined as:

(21) $$e_{k}=x_{k}-{\overset{´}{x}}_{k/k},$$

where $x_{k}$ is the true state, ${\overset{´}{x}}_{k/k}$ is the Kalman-filtered estimate. The Kalman estimation error covariance $P_{k}$ is [36]:

(22) $$P_{k}=E\left[e_{k}{e_{k}}^{T}\right],\;P_{k}=\left[\begin{array}{cc} P_{11} & P_{12}\\ P_{21} & P_{22} \end{array}\right],$$

Incorporating quantization noise, the LQG closed-loop state equation is:

(23) $$x_{k+1}=({A-BL_{LQR})x}_{k}+BL_{LQR}e_{k}+B\varepsilon_{k}+\omega_{k},$$

The closed-loop state covariance matrix $\Sigma_{k}$ can be given by:

(24) $$\;\Sigma_{k}=E\left[\left(x_{k}-E\left[x_{k}\right]\right){\left(x_{k}-E\left[x_{k}\right]\right)}^{T}\right],\;\Sigma_{k}=\left[\begin{array}{cc} \Sigma_{11} & \Sigma_{12}\\ \Sigma_{21} & \Sigma_{22} \end{array}\right],$$

The recursive formula for $\Sigma_{k}$ is:

(25) $$\Sigma_{k+1}=\left(A-BL_{LQR}\right)\Sigma_{k}{\left(A-BL_{LQR}\right)}^{T}+BL_{LQR}P_{k}{L_{LQR}}^{T}B^{T}+B{\Sigma_{\varepsilon }B}^{T}+Q$$

As shown in Equation (25), closed-loop errors originate from four parts, as follows:

$\left(A-BL_{LQR}\right)\Sigma_{k}{\left(A-BL_{LQR}\right)}^{T}$ is state propagation.

$BL_{LQR}P_{k}{L_{LQR}}^{T}B^{T}$ is estimation error representing Kalman filter error amplified by LQR gain.

$B{\Sigma_{\varepsilon }B}^{T}$ is quantization noise.

Q is process noise.

At steady state ($\Sigma_{k+1}=\Sigma_{k}=\Sigma_{\infty }$), the closed-loop pole λ (maximum eigenvalue magnitude), which determines the control stability, is defined as:

(26) $$\lambda =max\left|eig(A-BL_{LQR})\right|,$$

For a stable system λ<1, [37], $\Sigma_{11}$ and $\Sigma_{22}$ are derived as:

(27) $$\Sigma_{11}=\frac{Q_{11}+\tau^{2}\Sigma_{22}}{1-\lambda^{2}},\;\lambda =max\left|eig(A-BL_{LQR})\right|<1,$$

(28) $$\Sigma_{22}=\frac{{L_{1}}^{2}Q_{11}+(1-\lambda^{2})\left({L_{1}}^{2}P_{11}+{L_{2}}^{2}P_{22}+\Sigma_{\varepsilon }+Q_{22}\right)}{\left(1-\lambda^{2}\right)\left(1-{\left(1-L_{2}\right)}^{2}\right)-{L_{1}}^{2}\tau^{2}},$$

As shown in Equations (27) and (28), $\Sigma_{11}$ is driven by process noise and frequency deviation variance, while $\Sigma_{22}$ depends on control gains $\left[L1,\;\;L2\right]$, Kalman estimation errors ($P_{11}$, $P_{22}$), quantization noise $\Sigma_{\varepsilon }$, and process noise *Q*.

### 3.3. Hierarchical Constraint Framework for Frequency Precision Steering

A four-dimensional constraint integrated (FDCI) framework is constructed based on real-time precision positioning service requirements. Priority-driven weight parameter iteration through the FDCI framework is performed to achieve collaborative optimization. System performance specifications are comprehensively optimized via the precision control through the derived control parameters.

**Figure 2:**
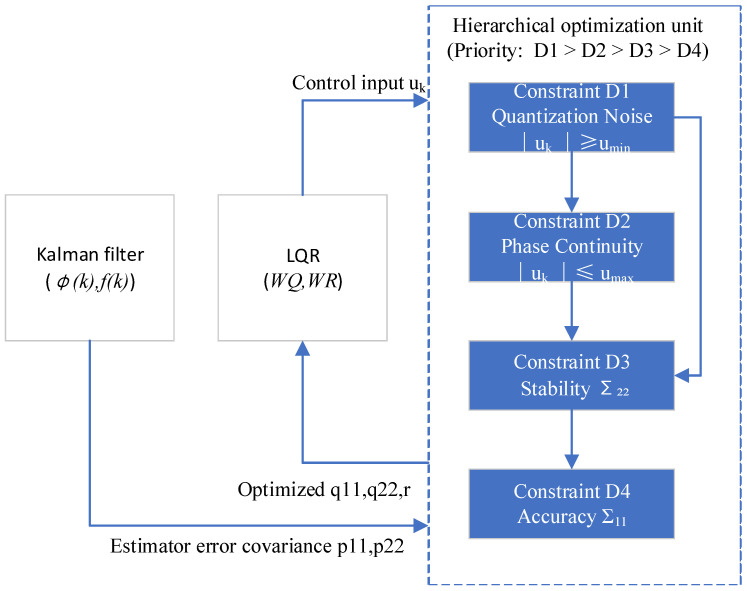
The block diagram of the FDCI model.

As shown in Figure 2, the FDCI model is established to translate real-time positioning requirements into actionable control constraints such as prioritizing quantization noise suppression (D1), phase continuity (D2), frequency stability (D3), and clock bias accuracy (D4). The model is operated through a hierarchical optimization unit (as depicted in Figure 1). Furthermore, the priority-driven sequence is D1 > D2 > D3 > D4. Strictly adhering to this priority, potential conflicts between the competing constraints are resolved by satisfying higher-priority constraints before optimizing lower-priority ones. Higher-priority constraints override lower-priority objectives through iterative tuning of the LQR weight matrices.

Firstly, constraint D1 is quantization noise suppression (highest priority), which limits the degradation in frequency stability caused by the finite resolution (quantization) of the frequency control mechanism. D1 limits the minimum frequency step $u_{min}$ to cap quantization-induced stability loss (Equation (31)). Constraint D1 ensures that the quantization noise introduced by the minimum control step does not dominate the oscillator’s inherent noise floor, preserving baseline stability.

Secondly, constraint D2 is phase continuity (second priority) that prevents the loss of lock in the receiver’s tracking loop. D2 limits the maximum rate of phase change induced by frequency adjustments. Constraint D2 ensures that the slew rate of the oscillator’s phase stays below a threshold that receiver tracking loops can accommodate without losing lock.

Thirdly, constraint D3 is frequency stability (third priority) that maintains the frequency short- or long-term stability of the controlled oscillator within a specified bound, characterized by the Allan deviation (ADEV). Constraint D3 enforces Allan deviation bounds. Constraint D3 ensures the controlled oscillator’s frequency fluctuations over longer periods meet the system’s stability requirement, crucial for sustained positioning accuracy.

Finally, constraint D4 is accuracy (lowest priority) that achieves the required positioning accuracy by bounding the root mean square (RMS) of the clock bias error that controls clock bias RMS.

The FDCI model provides a formal, mathematical framework (D1–D4) for incorporating critical oscillator limitations (quantization, phase slew, stability, bias) into an optimal control problem. The derivations of each constraint formula are presented in the following sections.

#### 3.3.1. Constraint D1 for Quantization Noise

For an open-loop or weakly controlled state, $Q_{22}$ and $\Sigma_{\varepsilon }$ are independent. Under a high-precision Kalman filter, $\Sigma_{22}$ can be expressed approximately as follows:

(29) $$\Sigma_{22}\approx Q_{22}+\Sigma_{\varepsilon },$$

Note: This approximation is validated under high-precision Kalman filtering conditions, where the inequality ${L_{2}}^{2}P_{22\ll }Q_{22}$ holds. Monte Carlo simulations demonstrate that the induced Allan variance calculation error is constrained to <5% for τ > 10 s, with such deviations being fully absorbed by the K-factor design margin. For short-term stability analysis (τ < 10 s), direct adoption of Equation (28) is recommended.

For ultra-short-term OCXO clock state models, the process noise for frequency deviation is dominated by white noise. The relationship between $\Sigma_{22}$ and oscillator inherent Allan variance ${\sigma_{osc}}^{2}(\tau )$ is:

(30) $${\sigma_{osc}}^{2}(\tau )=\frac{\Sigma_{22}}{2\tau^{2}},$$

Quantization noise, which degrades the short-term frequency stability, must be constrained to *K* times the inherent frequency stability. The constraint D1 for the minimum frequency step $u_{min}$ is established:

(31) $$\frac{{u_{min}}^{2}}{12}\leq {2\tau^{2}\left(K\sigma_{osc}\left(\tau \right)\right)}^{2}(upper\;bound)$$

where K is denoted as a scale factor that quantifies stability losses, with empirically constrained values of 0.10 to 0.30.

To ensure feasible micro-step actuation, the practical lower bound for D1 is as follows:

(32) $$u_{min}\geq 0.1\times \sigma_{osc}\left(\tau \right)\;(lower\;bound)$$

The control weight $W_{R}$ can be increased to suppress injected quantization noise.

#### 3.3.2. Constraint D2 for Phase Continuity

During frequency adjustments, the RF signal phase must be changed slowly and continuously, avoiding stepwise phase shifts or fluctuations. The navigation RF signal frequency $f_{RF}$ and the reference frequency $f_{ref}$ can be written:

(33) $$f_{RF}=N{\cdot f}_{ref},$$

where N is the frequency multiplication factor.

The phase-change rate $\frac{d\emptyset \left(t\right)}{dt}$ induced by the maximum frequency adjustment $u_{max}$ is:

(34) $$\frac{d\emptyset \left(t\right)}{dt}=2\pi \cdot N{\cdot f}_{ref}\cdot u_{max},$$

To ensure receiver tracking stability, the maximum tolerable phase rate of change is ${d\emptyset /dt}_{max}$; thus, the constraint D2 for phase continuity is given by:

(35) $$2\pi \cdot N{\cdot f}_{ref}\cdot u_{max}\leq {\left(\frac{d\emptyset \left(t\right)}{dt}\right)}_{max},$$

#### 3.3.3. Constraint D3 for Stability

Under the LQG closed-loop control, the frequency stability specification ${\sigma^{2}}_{y\_spec}\left(\tau \right)$ is the variance of the mean frequency deviation over a certain time window τ. The constraint D3 for frequency stability is given by:

(36) $${\sigma^{2}}_{y\_spec}\left(\tau \right)=\frac{\Sigma_{22}}{\tau },$$

where τ is the time window, typically ranging from 10 s to 300 s.

To prevent over-optimization beyond atomic clock reference limits, the performance requirement is as follows:

(37) $$\sigma_{y_{spec}}\left(\tau \right)\geq 0.5\times \sigma_{y_{atomic}}\left(\tau \right),$$

where $\sigma_{y_{atomic}}\left(\tau \right)$ is determined by the reference clock, for better frequency stability.

#### 3.3.4. Constraint D4 for Accuracy

The closed-loop clock bias variance $\Sigma_{11}$ indicates the system’s capability of controlling clock bias errors. The closed-loop clock bias error specification is $\delta_{max}$. The constraint D4 for accuracy is given by:

(38) $$\Sigma_{11}=\frac{Q_{11}+\tau^{2}\Sigma_{22}}{1-\lambda^{2}}\leq {\delta_{max}}^{2},$$

where λ is the dominant closed-loop pole magnitude, which must be less than 1.

Equation (38) relates bias variance (Σ_11_) to frequency variance (Σ_22_) and the closed-loop system dynamics (λ), ensuring the overall timing error RMS stays below the threshold.

To avoid over-damping natural oscillator behavior, the dynamical preservation bound is enforced:

(39) $$\Sigma_{11}\geq 0.1\times Q_{11}$$

#### 3.3.5. Four-Dimensional Constraint Integrated (FDCI) Model Parameters

According to Section 3.3.1, Section 3.3.2, Section 3.3.3 and Section 3.3.4, constraints D1 and D2 directly bound the control input magnitude ($u_{min}$, $u_{max}$). Constraints D3 and D4 act on state variances (Σ_22_, Σ_11_) predicted by the Kalman filter state estimator using the system model and measurements (observations from a GNSS receiver).

For real-time centimeter-level position, FDCI model parameters are mathematically formalized in Table 1.

By rigorously constraining quantization noise (D1), ensuring phase continuity for receiver tracking (D2), guaranteeing long-term stability (D3), and directly bounding the clock bias RMS (D4), the FDCI model is fundamental to achieving and sustaining real-time centimeter-level positioning accuracy. The specific D4 constraint (Σ_11_ ≤ (0.15 ns)^2^) directly targets the required positioning accuracy at 4.5 cm (represented by 0.15 ns × c, where c is 3 × 10^8^ m/s).

### 3.4. Priority-Driven Co-Optimization Algorithm Flow

The LQR gain $L_{LQR}$ is derived from $W_{Q}$ and $W_{R}$ via the Riccati equation. The priority is D1 > D2 >D3 > D4. The priority-driven hierarchical optimization process is implemented as follows:

#### 3.4.1. Initialization

1. Oscillator noise characterization

Oscillator noise types are analyzed through Allan variance measurements. The process noise covariance matrix *Q* is constructed from the identified noise characteristics.

2. Adjustment range set

The minimum frequency step $u_{min}$ is determined based on quantization noise constraints (constraint D1). The maximum adjustment threshold $u_{max}$ is subsequently established using phase continuity requirements (constraint D2).

#### 3.4.2. Iterative Optimization

Iterative weight parameter optimization is achieved via adjustment factors α, *β*, and γ, assigned to $q_{11}$, $q_{22},$ and *r*, respectively.

1. Stability constraint enforcement (D3)

Long-term frequency stability is optimized by:

- The frequency deviation weight *q*_22_ being increased by factor α;
- The control input weight r being decreased by factor *β*;
- This adjustment sequence is repeated until the stability constraint is satisfied.

2. Accuracy constraint enforcement (D4)

Clock bias variance is minimized by:

- The clock bias weight *q*_11_ being increased by factor γ;
- The control weight r being slightly incremented *β*_1 for control smoothing;

Constraint D4 satisfaction is verified after each adjustment

3. Adjustment step safeguarding

During each iteration, the theoretical adjustment $u_{k}$ is computed using current LQG parameters and the magnitude ∣$u_{k}$∣ is compared against $u_{max}$. If exceeded:

- $u_{k}$ is clamped to $u_{max}$;
- $q_{22}$ is decreased;
- *r* is increased;
- The stability optimization step is reinitiated.

4. Convergence verification

Termination criteria are evaluated after constraint satisfaction:

- Parameter variations < 0.1% over three consecutive iterations;
- Closed-loop pole magnitude λ < 0.95;

If unmet, the optimization loop is re-entered.

5. Command execution

Adjustment quantization is that the optimized frequency adjustment $u_{k}$ is quantized to $u_{k}^{*}$.

6. System actuation

Executable frequency control commands are generated and transmitted to the oscillator actuation unit.

#### 3.4.3. Process of the Parameter Optimization Algorithm

**Figure 3:**
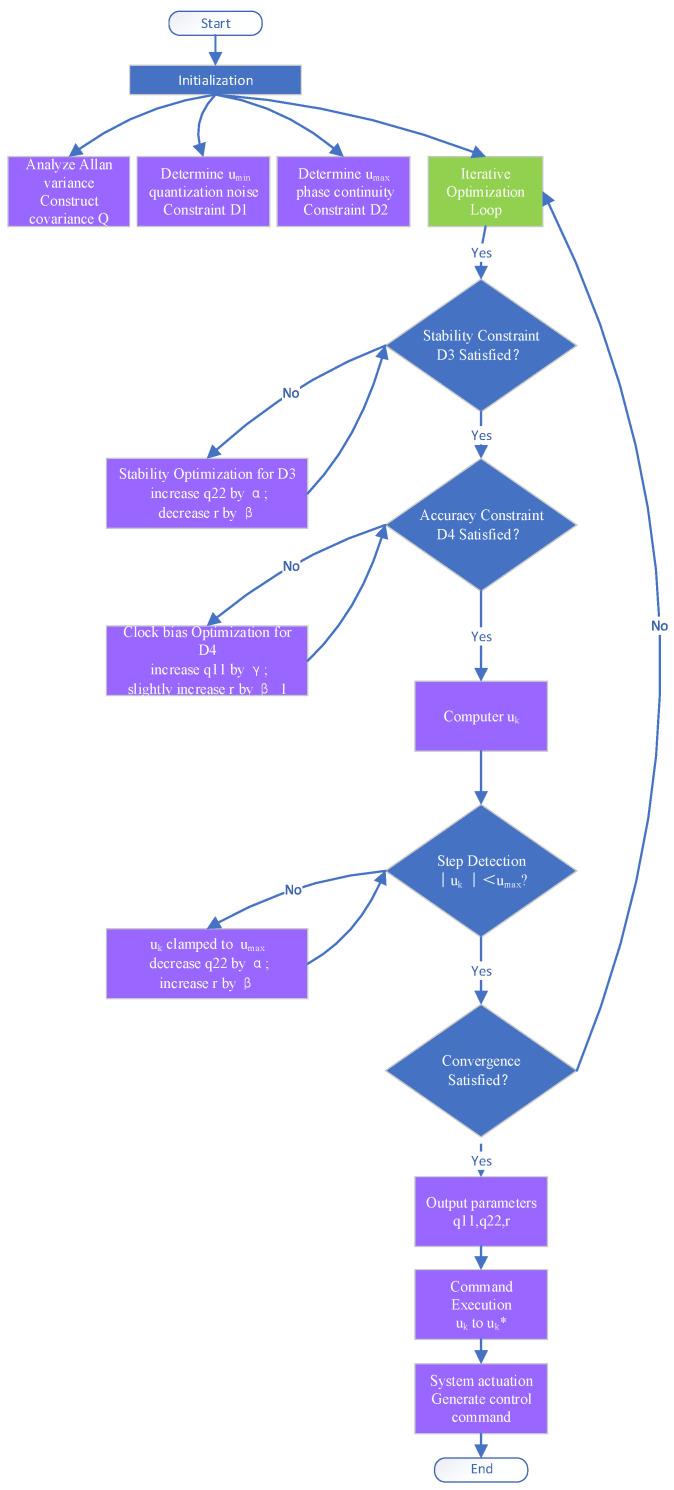
The flowchart of the priority-driven co-optimization algorithm.

Based on the algorithm description given above, the iterative optimization logic is visualized as illustrated in Figure 3.

Firstly, D1 is enforced by ensuring the magnitude of any commanded step respects $\left|u_{k}\right|\geq u_{min}$.

Secondly, D2 is enforced by clipping the commanded step to $\left|u_{k}\right|\leq u_{max}$.

Thirdly, D3 is enforced by verifying the control input or tuning LQR weights such that the estimated frequency variance *Σ*_22_ meets the ADEV bound.

Fourthly, D4 is enforced by verifying the control input or tuning LQR weights such that the estimated clock bias variance Σ_11_ meets the RMS bias bound.

Multi-constraint conflicts (e.g., D1 quantization noise vs. D4 accuracy) are systematically resolved through the hierarchy priority (D1 > D2 > D3 > D4). Iterative weight tuning prioritizes quantization suppression first (D1), followed by phase continuity (D2), enabling sequential relaxation of lower-priority constraints (D3, D4). This ensures Kalman estimation errors and LQR gains are co-optimized without empirical compromises, as validated in Section 4.2.

Especially, if lower-priority constraints (D3/D4) cannot be satisfied without violating a higher-priority constraint (D1/D2), the higher-priority constraint prevails. Iterative tuning of the weights (*q*_11_, *q*_22_, *r*) is a key mechanism to resolve conflicts and find feasible control inputs satisfying the prioritized constraint hierarchy.

Through this multi-constraint collaborative optimization, the algorithm achieves an optimal balance between time–frequency stability and accuracy.

## 4. Experiment Validation

### 4.1. Architecture of the High-Precision Time–Frequency Control System

A rubidium atomic clock is utilized as the time–frequency reference to perform real-time frequency steering on OCXOs.

**Figure 4:**
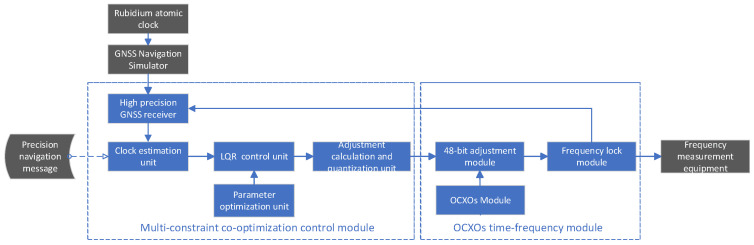
Block diagram of the LQG frequency-controlled system with multi-constraint collaborative optimization.

The complete system architecture was illustrated in Figure 4. The reference rubidium clock was operated at 10 MHz with frequency accuracy of 2.0 × 10^−12^ and frequency drift of 1.66 × 10^−13^. The OCXO (model RK409, Rakon Inc., Auckland, New Zealand) was also operated at 10 MHz and had initial frequency accuracy of 3.5 × 10^−9^. The frequency short-term stability is 4.5 × 10^−13^ at 1 s, 6.1 × 10^−13^ at 10 s, and 1.1 × 10^−12^ at 100 s. The frequency long-term stability is 8.2 × 10^−11^ per day. The stability versus temperature sensitivity is ±0.1 ppb.

The GNSS signal simulator generated simulated navigation signals based on precise GNSS satellite ephemerides, clock biases, and LEO satellite orbits. A high-precision GNSS receiver obtained pseudorange and carrier-phase observations at a rate of 1 Hz for the determination of local clock bias. A Kalman filter integrated these observations with precise orbit and clock corrections to estimate the optimal clock bias and frequency deviation, enhanced by incorporating precise LEO orbital information [36].

The parameter optimization unit carried out collaborative optimization of the weighting matrices $W_{Q}$ and $W_{R}$ under multiple constraints, determining optimal weight design values. Frequency steering was triggered whenever the clock bias exceeded the threshold (0.15 ns). The steering calculation unit computed frequency adjustment values through LQR based on optimized weight matrices. These adjustments were quantized within predefined constraints, and frequency control information was injected into the OCXO system for precise frequency adjustment.

System workflow is described as follows:

1. GNSS Signal Simulation: A GNSS signal simulator generated navigation signals using precise ephemerides, satellite clock biases, and LEO precise orbital data.
2. Clock Bias Measurement: A high-precision GNSS receiver acquired pseudorange and carrier phase observations at 1 Hz to determine local clock bias.
3. State Estimation: A Kalman filter fused GNSS observations with injected orbit/clock data to estimate clock bias $\varphi \left(k\right)$ and frequency deviation $f\left(k\right)$. LEO orbital corrections were applied to enhance accuracy [38].
4. Parameter Optimization: The control module optimized weight matrices $W_{Q}$ and $W_{R}$ under multi-constraint conditions.
5. Steering Trigger: Frequency adjustment was initiated when clock bias exceeded ±0.2 ns.
6. Command Execution: The steering unit calculated $u_{k}$, quantized it to $u_{k}^{*}$ (subject to $u_{min}{\leq u}_{k}{\leq u}_{max}$), and sent commands to the OCXO.
7. Performance Evaluation: Output signals were monitored for 24 h to measure clock bias and frequency stability.

The effectiveness of the frequency steering method was evaluated through continuous experiment operation for 24 h.

### 4.2. Results of LQG Parameter Optimization Under Multi-Constraint

The ranges of weight matrices were defined through initial scanning as follows:

- $q_{11}$ in $W_{Q}$ was constrained within 1 × 10^−1^ to 1 × 10^3^
- $q_{22}$ in $W_{Q}$ was bounded between 1 × 10^1^ and 1 × 10^4^
- r was limited to the interval 1 × 10^3^ to 1 × 10^10^.

**Figure 5:**
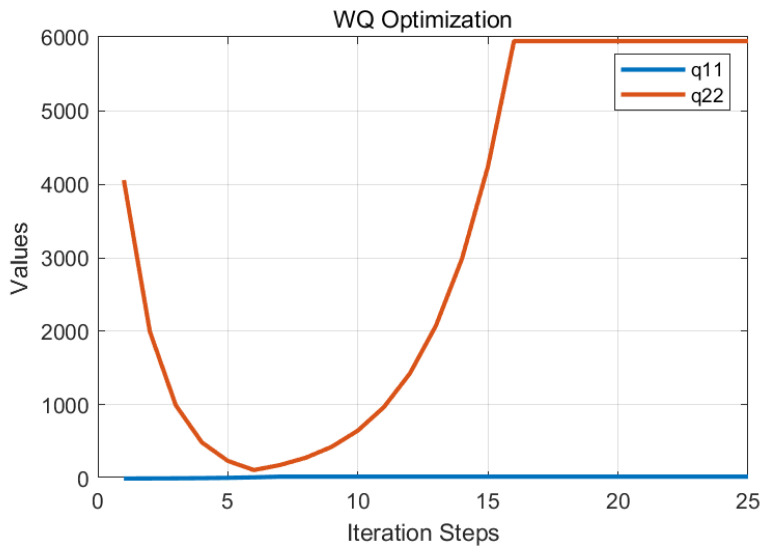
Weight matrix parameters $W_{Q}$ optimization process.

**Figure 6:**
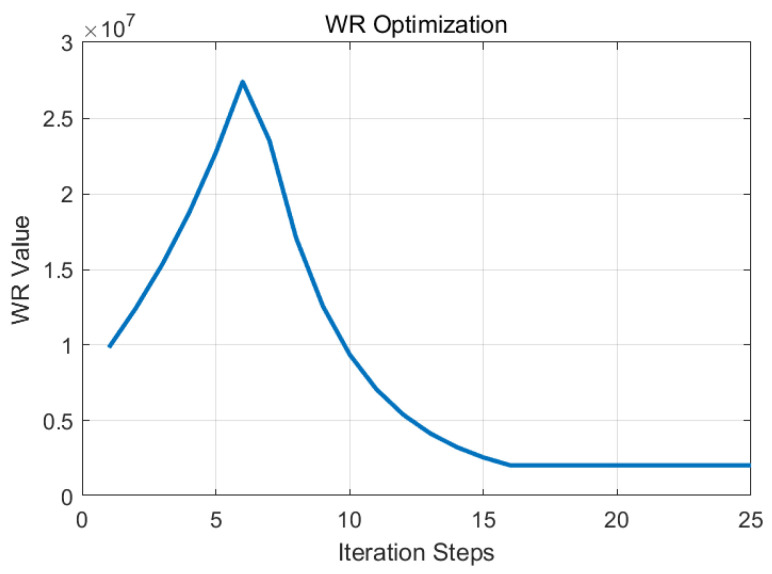
Weight matrix parameters $W_{R}$ optimization process.

Through adjustment factors α, β, β_1, and γ, a hierarchical collaborative optimization of parameters $q_{11}$, $q_{22}$, and *r* was performed as shown in Figure 5 and Figure 6. Following 15 iterations, the optimal parameters were established as $q_{11}$ = 23.4, $q_{22}$ = 5940, and *r* = 2.1 × 10^6^, with corresponding feedback gain matrices computed at $L_{1}$ = 3.2 × 10^−3^ and $L_{2}$ = 9.65 × 10^−2^.

**Figure 7:**
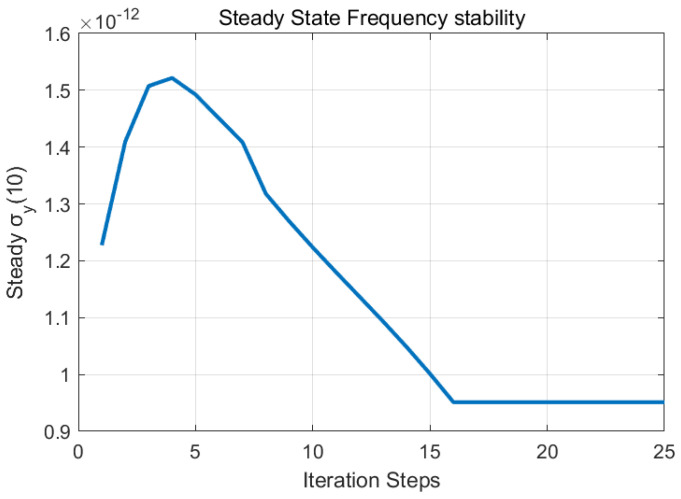
Closed-loop system frequency stability convergence under constraint.

**Figure 8:**
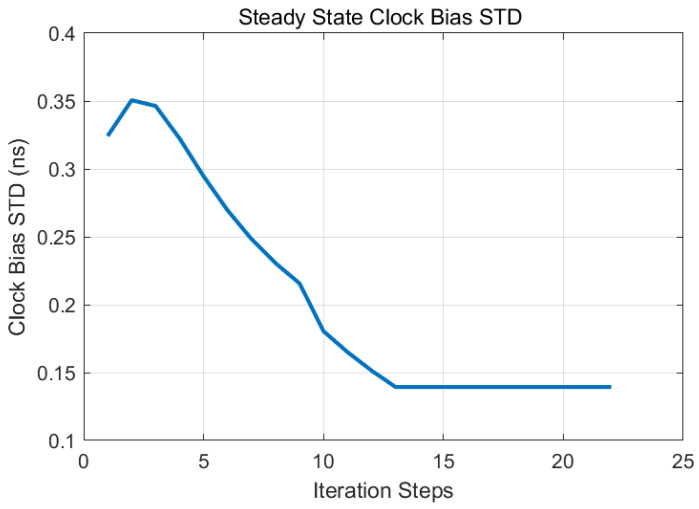
Closed-loop system clock bias accuracy convergence under constraint.

Steady-state performance is illustrated in Figure 7 and Figure 8. The steady-state clock bias accuracy was 0.14 ns, with a frequency stability of 9.5 × 10^−13^ at 100 s, meeting optimal performance criteria.

### 4.3. Frequency Adjustment Analysis

**Figure 9:**
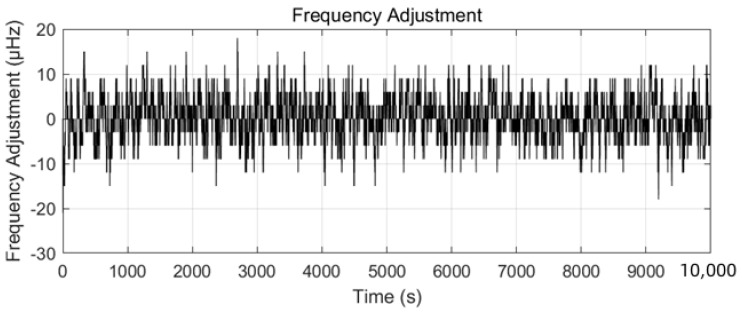
Frequency adjustments under optimized LQG control.

Based on the optimized $W_{Q}$ and $W_{Q}$ from Section 4.2, frequency adjustments were bounded within ±15 µHz (5 × $u_{min}$ relative to 10 MHz), as depicted in Figure 9.

Phase-change rates remained below 0.1 rad/s, satisfying receiver tracking limits.

### 4.4. System Performance Under the Optimized LQG Control

Performance of clock bias accuracy and frequency stability were conducted for the time–frequency product under the optimized LQG control.

#### 4.4.1. Measurement Results for Clock Bias Performance

**Figure 10:**
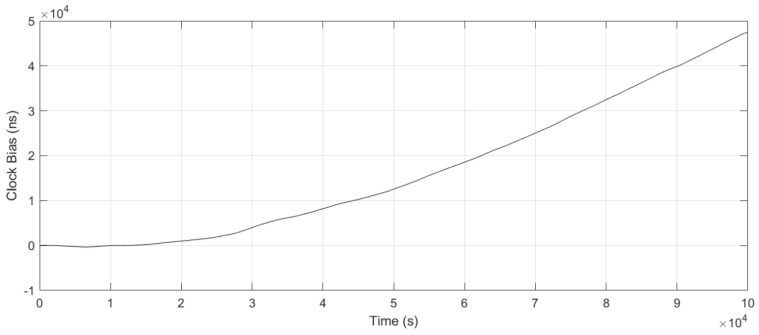
Free-running clock bias.

**Figure 11:**
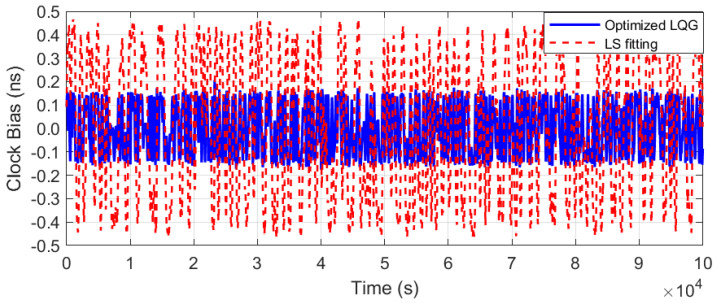
Clock bias comparison with optimized LQG control and LS fitting.

**Figure 12:**
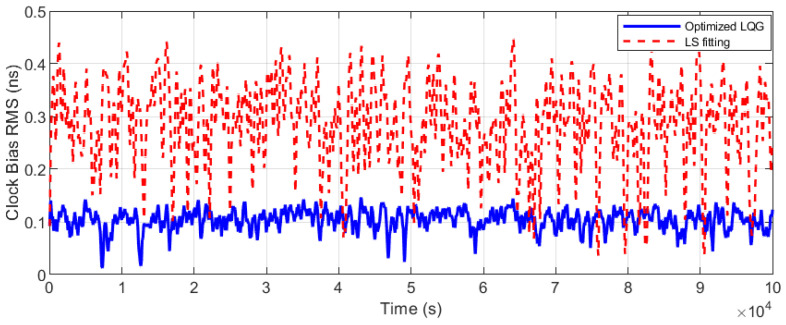
Clock bias accuracy (RMS) comparison with optimized LQG control and LS fitting.

Figure 10 shows the OCXO’s clock bias with free-running, rapidly accumulating to 930 ns after 20,000 s, reflecting a drift rate of 170 ns/h. After applying the optimized LQG steering method, as depicted in Figure 11 and Figure 12, the clock bias fluctuation (peak to peak) was maintained within 0.3 ns, achieving the accuracy (RMS) of 0.15 ns.

In contrast, the unconstrained least-squares (LS) fitting for frequency correction was conducted. Due to static compensation limitations, the unconstrained LS method exhibits a peak-to-peak clock bias fluctuation of 0.93 ns (red curve in Figure 11, labeled ‘LS Fitting’). Additionally, 0.46 ns RMS accuracy was achieved, as shown in Figure 12 (red height), which is constrained by the fixed polynomial model’s inability to track dynamic changes.

Positioning accuracy is directly determined by RMS performance, and a 67.7% improvement was demonstrated by the optimized LQG over the LS method.

Furthermore, a comparative analysis was conducted against the multi-source data fusion strategy [17], which integrates GNSS inter-satellite links and ground references. For a one-hour prediction period, the clock error through data fusion achieved 0.25 ns, while the optimized LQG reached 0.14 ns, demonstrating a 44% improvement.

The LQG approach’s real-time micro-step actuation (1 Hz) outperforms fusion in responsiveness and stability, as fusion’s slow updates induce phase discontinuities.

#### 4.4.2. Measurement Results for Frequency Deviation

**Figure 13:**
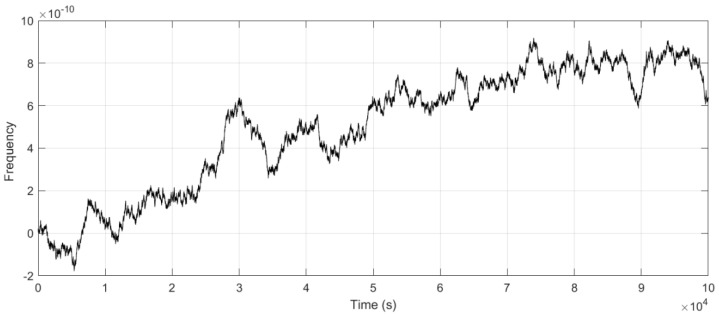
Free-running frequency deviation.

**Figure 14:**
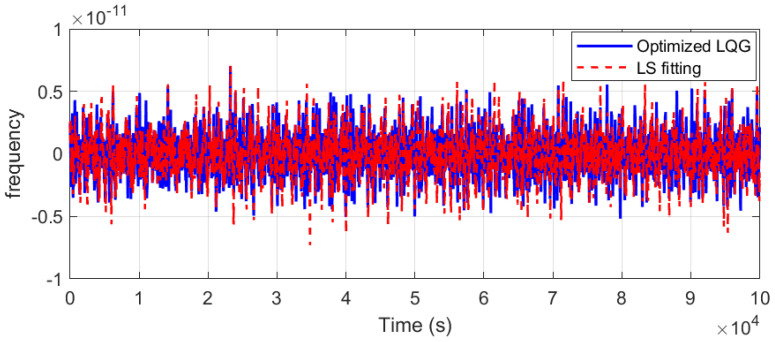
Frequency deviation comparison with optimized LQG control and LS fitting.

The frequency of the time–frequency signal was tested. As shown in Figure 13, under free-running conditions, the OCXO exhibited an initial frequency accuracy of 2.3 × 10^−11^. Due to long-term frequency drift, the accuracy degraded to 7.36 × 10^−11^ after 30,000 s. After implementing the optimized LQG control, the frequency accuracy shown in Figure 14 indicates that post-adjustment fluctuations were constrained to the 2.5 × 10^−12^ level. These results demonstrate that the steering method effectively suppressed the oscillator’s random walk characteristics. The adjusted frequency curve exhibited near-zero-mean Gaussian white noise properties, improving accuracy by two orders of magnitude compared to the free-running state.

Upon the frequency deviation presented in Figure 14 (red curve), the LS fitting method was observed to achieve a ranging between approximately 7 × 10^−12^ and 8.9 × 10^−12^.

Consequently, the optimized LQG method significantly outperformed the LS approach by 72% in suppressing random walk noise and achieving near-zero-mean Gaussian white noise characteristics.

#### 4.4.3. Measurement Results for Frequency Stability

**Figure 15:**
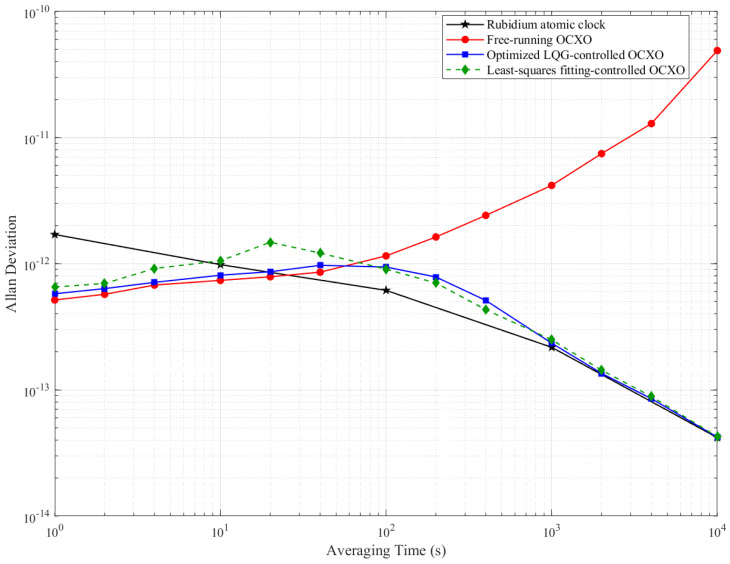
Frequency stability comparison with reference rubidium clock, free-running OCXO, and optimized LQG OCXO.

Comparative evaluations of frequency stability (Allan deviation, ADEV) were performed among the reference rubidium clock, free-running OCXO, optimized LQG-controlled OCXO, and unconstrained least-squares (LS) fitting OCXO, as shown in Figure 15.

Note: The log–log plot in Figure 15 clearly visualizes stability tradeoffs (1–100 s for short-term, 100–400 s for short-long term, and >400 s for long-term).

Due to quantization noise in the steering process, a slight degradation in short-term stability (1–40 s) was observed in the optimized LQG-controlled oscillator compared to the free-running state. Despite up to 13% stability degradation at 40 s (max 9.68 × 10^−13^), the achieved short-term stability at 1–40 s averaging times still satisfies the tolerance for centimeter-level positioning requirements.

As shown in Equation (31), the stability degradation is constrained by K for LEO GNSS applications; stability thresholds for centimeter-level accuracy are permitted up to the 10^−12^ level [39]. The 9.68 × 10^−13^ achieved at 40 s induces only 0.04 ns additional clock estimation error, equivalent to a positioning error of 1.2 cm (δ = τ × $\sigma_{y}\left(\tau \right)$). This is negligible for centimeter-level services when the threshold of the clock bias error is 0.3 ns.

Obvious improvements in frequency stability were demonstrated at averaging intervals exceeding 40 s. Crucially, in the 100–400 s interval, which serves as a transition to long-term stability, the optimized LQG method exhibited consistent improvement over free-running operation. At 200 s, the Allan deviation was improved from 1.15 × 10^−12^ of free-running to 9.38 × 10^−13^ under optimized LQG control. Beyond 400 s, significant enhancement was achieved from the 10^−12^ level to 10^−13^ level. At 1000 s and 10,000 s, the mid- to long-term stability reached parity with the rubidium atomic clock performance.

In contrast, the application of unconstrained least-squares (LS) fitting for frequency correction was conducted with long-term frequency drift effectively mitigated. However, substantial degradation in short-term frequency stability was introduced by this correction process, specifically within the 1–40 s range.

Especially, the stability characteristics are quantitatively supported by the Allan deviation measurements shown in Table 2. Improvement ratios are provided to facilitate direct comparison between the free-running baseline and optimization approaches.

As presented in Table 2, the loss of stability under optimized LQG control was up to 13% at 40 s. Frequency stability parameters under the LS fitting correction deteriorated by up to 88% in the 1–40 s range overall, with particularly severe impacts noted at specific intervals: instability was increased by 43% at 10 s, 88% at 20 s, and 42% at 40 s, compared to free-running operation.

Finally, the LQG method maintains near-optimal stability from 1 s to 10,000 s, with a maximum 13% degradation, significantly lower than the 88% degradation under LS fitting.

#### 4.4.4. Comprehensive Improvement Analysis for Performance Metrics

As quantified in Table 3, substantial performance enhancements across critical time–frequency metrics are demonstrated by the optimized LQG method. The following systematic analysis reveals the superiority over conventional LS approaches.

Firstly, clock bias peak-to-peak was reduced to 0.3 ns, representing 99.97% improvement versus free-running OCXO and a 66.67% improvement from LS fitting.

Similarly, clock bias RMS was compressed to 0.14 ns (positioning accuracy 4.2 cm), representing a 69.6% improvement from LS fitting benchmarks, effectively transforming positioning precision from meter-level to centimeter-level.

In addition, frequency deviation was suppressed to 2.5 × 10^−12^ (clock bias drift rate 0.0025 ns/s), demonstrating 72% improvement over LS methodology.

Critical mid-term stability at 100 s achieved 9.38 × 10^−13^, maintaining near parity with LS fitting (3.9% difference).

Long-term stability at 1000 s was enhanced to 2.34 × 10^−13^, representing 94.39% improvement over free-running oscillators and 6% advancement beyond LS fitting.

Through quantifiable optimization, the optimized LQG method demonstrated overwhelming advantages in six of seven key metrics, as shown in Table 3, with particular efficacy in long-term drift suppression evidenced by 99.91% stability improvement at 10,000 s. The optimized LQG method was demonstrated to outperform the LS approach especially in positioning accuracy, which is determined by clock bias error.

### 4.5. Environmental Robustness Validation of the Optimized LQG Control

To assess the space environmental adaptability of the control method, the robustness of the control system was quantitatively evaluated through thermal vacuum and vibration tests. The robust results were correlated with the performance metrics shown in Section 4.4.4 (optimized LQG) for comprehensive analysis.

#### 4.5.1. Experimental Design

Firstly, the thermal vacuum experiment was conducted to simulate the in-orbit thermal environment and validate the method’s adaptability under vacuum and extreme thermal stress. The test conditions were defined as follows:

- Vacuum level: better than 6.5 × 10^−3^ Pa;
- Heat sink temperature: not exceeding 100 K;
- Temperature gradient range: from −20 °C to +45 °C at 10 °C intervals;
- Temperature stability requirement: ±1.5 °C;
- Dwell time per temperature point: 6 h;

The test profile progressed sequentially from low to high temperatures: −20 °C, −10 °C, 0 °C, 10 °C, 25 °C, and 45 °C. Continuous data acquisition and performance evaluation were carried out throughout the 6 h dwell period at each stabilized temperature point.

Furthermore, random vibration and sinusoidal vibration tests were designed to simulate the in-orbit mechanical environment and validate the control method’s performance stability under sustained mechanical disturbances. The total duration for each vibration test type was set at 3 h. The first 1.5 h period (robustness assessment during vibration) was allocated for active vibration application. The subsequent 1.5 h period (performance monitoring post-vibration) was dedicated to evaluate recovery characteristics. To prevent product overstress damage and more realistically simulate disturbance scenarios, vibration was applied intermittently during the excitation phase.

1. Random Vibration Test Conditions
  - Frequency range: 20 Hz to 2000 Hz;
  - Acceleration power spectral density (PSD): +3 dB/oct from 20 Hz to 100 Hz, 0.02 g^2^/Hz from 100 Hz to 1000 Hz, −6 dB/oct from 1000 Hz to 2000 Hz;
  - Overall RMS level: 5.38 g_rms_;
  - Directional application: 2 min per axis (*X*, *Y*, *Z*);
  - During the active excitation phase, vibration was applied in 10 cycles. Each cycle consisted of one 6 min vibration followed by a 2 min pause.
2. Sinusoidal Vibration Test Conditions
  - Frequency sweep range: 5 Hz to 100 Hz;
  - Vibration amplitude: 3 mm (0 to peak) from 5 Hz to 12 Hz, 0.5 g from 12 Hz to 100 Hz.
  - Sweep rate: 4 oct/min;
  - Approximate sweep duration: 65 s;
  - During the active excitation phase, vibration was applied in 15 cycles. Each cycle consisted of one frequency sweep (65 s) followed by a 4 min pause.

Continuous data acquisition and test assessments were maintained throughout the entire 3 h duration for each vibration test type.

#### 4.5.2. Thermal Impact Analysis

To evaluate the control system’s adaptability to extreme in-orbit thermal environments, multi-temperature-point analysis of performance metrics was performed under vacuum conditions, as specified in Section 4.5.1.

**Figure 16:**
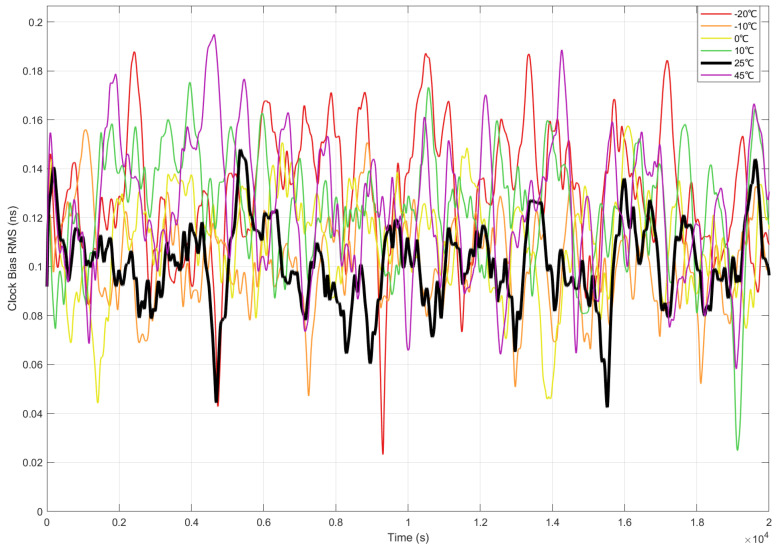
Clock bias accuracy (RMS) comparison with reference temperature (25 °C).

The clock bias error (RMS) across thermal conditions (setpoints from −20 °C to +45 °C), referenced to the 25 °C baseline, is compared in Figure 16. Under extreme temperatures (−20 °C and +45 °C), significant error fluctuations were observed, with peak clock bias error reaching 0.19 ns (e.g., red −20 °C curve), representing a 35% volatility increase versus the 25 °C baseline. In contrast, the 0 °C to −10 °C range maintained minimal error fluctuations (≤0.02 ns), with the 0 °C profile (yellow curve) exhibiting near-baseline level. The 25 °C reference recorded consistently optimal performance (0.14 ns), confirming nominal operating conditions.

The effectiveness of the optimized LQG control was demonstrated by confining absolute clock bias errors to ≤0.19 ns, corresponding to positioning error ≤ 5.7 cm, while error fluctuation amplitude was constrained within 0.04 ns through adaptive weight optimization.

**Figure 17:**
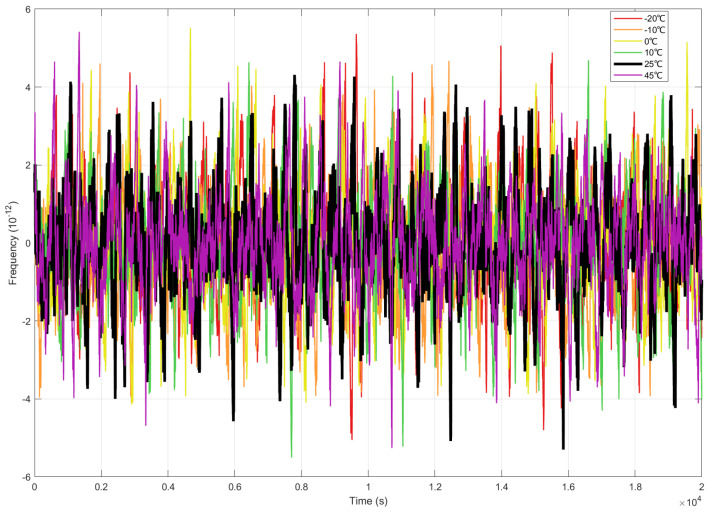
Frequency deviation comparison with reference temperature (25 °C).

As shown in Figure 17, significant frequency deviations relative to the reference temperature (25 °C) were observed across six thermal stress points (−20 °C to +45 °C).

The temperatures (−20 °C, 10 °C and +45 °C) showed the maximum frequency deviation at ±5.4 × 10^−12^ (e.g., the −20 °C red curve). All temperature profiles exhibited frequency deviations within ±6 × 10^−12^ (*Y*-axis range: −6 to +6, scaled to 10^−12^), with no systematic correlation between temperature and deviation direction.

Maximum observed frequency deviation still represented a 93% reduction versus free-running oscillators (>9.15 × 10^−11^, Section 4.4.2), confirming enhanced disturbance rejection capability. All frequency deviation profiles achieved attaining alignment within 30% of baseline performance.

**Figure 18:**
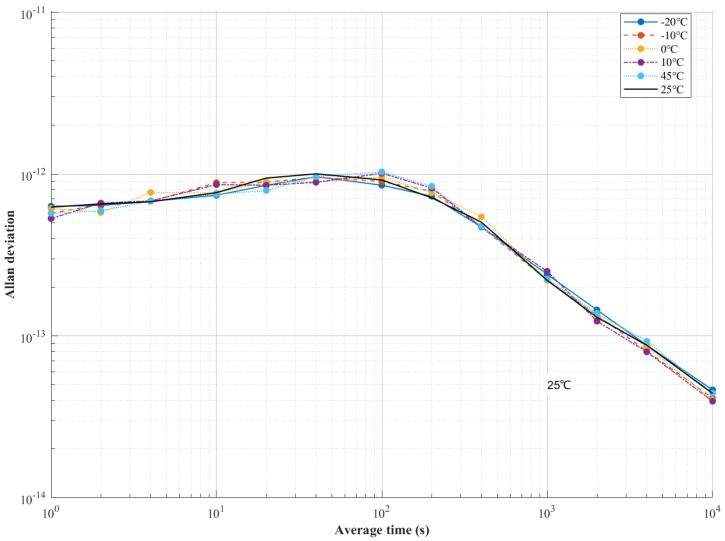
Frequency stability comparison with reference temperature (25 °C).

Frequency stability (Allan deviation, ADEV) across six temperature setpoints (−20 °C to +45 °C) is compared in Figure 18. ADEV curves were confined within a stability band of 1.2 × 10^−13^ to 4.3 × 10^−14^ across 1 s to 10,000 s.

At the critical interval (τ = 100 s), ADEV values were maintained between 1.03 × 10^−12^ (blue curve −20 °C) and 8.53 × 10^−13^ (light blue curve 45 °C), exhibiting ≤15% deviation from baseline. At interval 1000 s, ADEV was compressed to 2.2 × 10^−13^ ± 0.2 × 10^−13^ cross-temperature deviation, representing a 100-fold improvement versus uncontrolled oscillators (Table 2). Atomic clock-level stability (4 × 10^−14^) was achieved universally at τ = 10,000 s, where inter-temperature variation was suppressed to ≤0.2 × 10^−14^.

Under the optimized LQG control, temperature-dependent frequency drift was effectively eliminated, evidenced by consistent convergence to the ADEV baseline.

#### 4.5.3. Vibration Impact Analysis

To evaluate the control system’s adaptability to mechanical disturbances, comprehensive vibration performance analysis was conducted under random and sinusoidal excitation conditions, as specified in Section 4.5.1.

**Figure 19:**
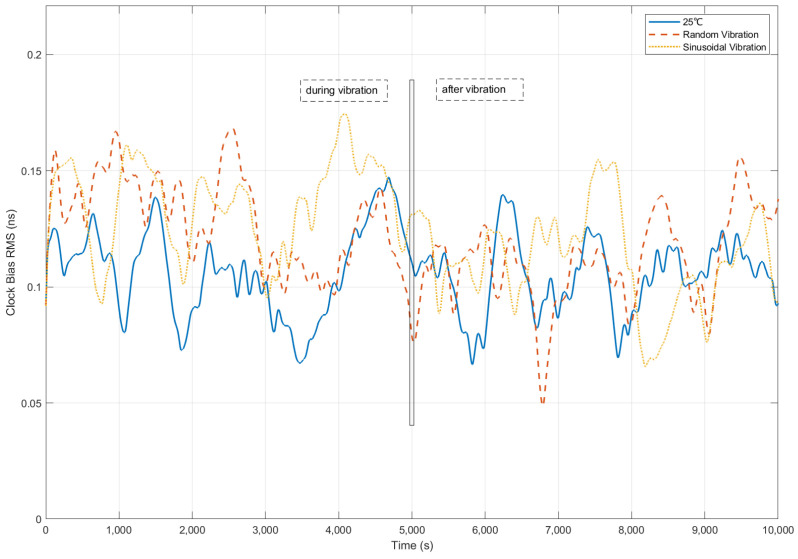
Clock bias accuracy (RMS) comparison under vibrations.

Clock bias error (RMS) across three vibrational regimes (non-vibration (solid blue line), random vibration (red dashed line), and sinusoidal vibration (yellow dash-dotted line)) is quantitatively compared in Figure 19 at 25 °C ambient temperature.

During the vibration, peak vibration-induced clock bias error was constrained to 0.17 ns across all conditions, 43% below the threshold of 0.3 ns (representing positioning error 10 cm). Moreover, sinusoidal vibration deviations were suppressed within 21% of non-vibration levels (0.17 ns vs. 0.14 ns), validating iteration efficacy against periodic disturbances.

Post-vibration recovery to baseline level (0.14 ± 0.02 ns) was achieved within 1000 s, as validated at 6000 s, confirming rapid stabilization of the proposed control method.

**Figure 20:**
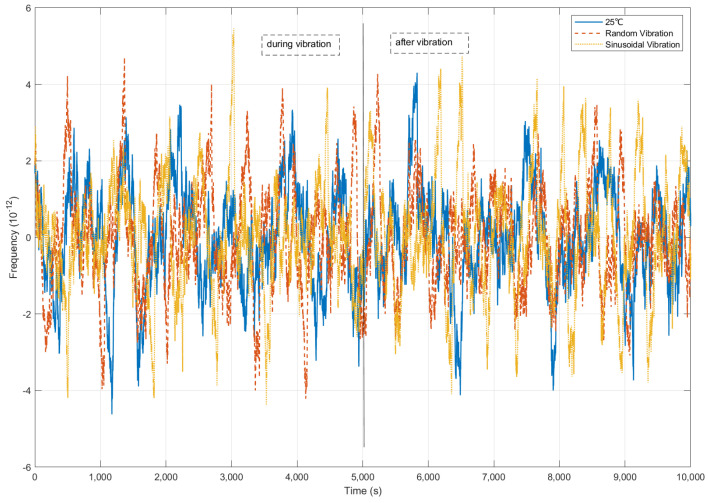
Frequency bias comparison under vibrations.

Frequency deviation under mechanical vibrations is rigorously characterized in Figure 20. During the vibration exposure phase, peak frequency deviation was 4.67 × 10^−12^ under random vibration, while sinusoidal vibration induced the peak at 5.45 × 10^−12^.

Despite these disturbances, deviation was constrained below the 1 × 10^−11^ threshold (representing clock bias drift rate 0.01 ns/s) through the frequency compensation. Post-exposure stabilization to 4.5 × 10^−12^ was achieved within 1000 s, with final-state restoration to 4.1 × 10^−12^. This confirms the controller’s capability to suppress vibrational noise and autonomously restore baseline performance.

**Figure 21:**
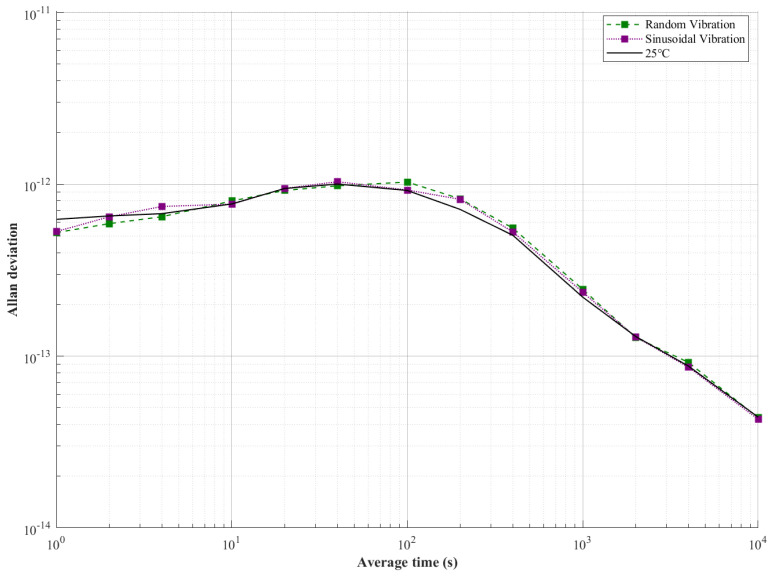
Frequency stability comparison under vibrations.

Frequency stability under mechanical disturbances is rigorously quantified in Figure 21 through comparative Allan deviation (ADEV) analysis across three conditions: 25 °C baseline (solid black), random vibration (green dashed), and sinusoidal vibration (purple solid).

Throughout the mechanical vibration exposure, Allan deviation metrics were maintained, converging with the reference baseline. Despite short-term stability fluctuations, ADEV was maintained within the 6–7 × 10^−13^ range, with variations confined to within 10% compared to the baseline. The mid-term stability (100–400 s) was dominated by vibration effects. Maximum degradation was observed at 200 s, where a frequency stability of 8.2 × 10^−13^ was recorded, exceeding the 25 °C baseline value (7.1 × 10^−13^) by 15%, while the long-term consistency (τ > 1000 s) was restored through convergence within ±0.1 × 10^−14^ at τ = 10,000 s, regardless of mechanical disturbance.

#### 4.5.4. Comprehensive Analysis for Robustness Results

The variance of clock performance across thermal and vibrational conditions is rigorously quantified in Table 4.

Critically, the clock bias RMS remains below 0.19 ns at +45 °C, corresponding to a positioning error of ≤5.7 cm. This represents 35% degradation relative to the 25 °C baseline, yet remains significantly below the 10 cm threshold for GNSS precision services.

Compared to the 25 °C baseline metrics, the worst-case frequency deviation was reduced to the 5.36 × 10^−12^–5.52 × 10^−12^ range, corresponding to a clock drift rate of 0.005 ns/s, with variations confined within 30%. A 20-fold stability improvement was achieved versus uncontrolled systems (9.5 × 10^−11^), as detailed in Table 3.

Frequency stability (Allan deviation) is proved more robust, with degradation constrained to ≤12% at 100 s and ≤5% at 10,000 s. Consistent drift suppression was demonstrated through ADEV metrics across all temperatures and vibrations, confirming elimination of environment-dependent long-term instability.

Consequently, the global robustness of the system is demonstrated through the bounded performance loss 35% (within design margins) and preserved operational long-term stability during extended testing.

## 5. Conclusions

A multi-constraint co-optimization framework integrating FDCI constraints and LQG control is established to resolve the inherent tradeoff between OCXO short-term stability and long-term drift. Firstly, quantitative assessment criteria (Table 1) for steering performance are introduced, eliminating the subjectivity in traditional empirical parameter tuning with mathematically formalized benchmarks. Secondly, multi-dimensional tradeoff balancing are resolved through a priority-driven constraint hierarchy optimization (D1> D2 > D3 > D4), enabling global optimization of clock bias, phase continuity, and stability. Thirdly, atomic-clock-grade stability is achieved at crystal-oscillator cost via micro-step actuation, representing the 0.3 ns timekeeping in LEO dynamic environments.

Comprehensive experimental results demonstrate that the proposed approach’s dual capability preserves excellent inherent short-term frequency stability while significantly enhancing accuracy and long-term frequency stability. Specifically, the clock error (RMS) is reduced to 0.14 ns, representing 37% improvement versus multi-source data fusion and 69.6% versus LS. Furthermore, the frequency deviation is reduced to 2.5 × 10^−12^, representing a 37-fold improvement over free-running operation. Crucially, the long-term frequency stability at 10,000 s has been improved to 4.22 × 10^−14^, achieving three-orders of magnitude enhancement compared to the free-running oscillator. Despite up to 13% stability degradation at 40 s (attributed to quantization), the optimized LQG approach maintains balanced stability across all intervals.

The real-time precision frequency correction for centimeter-level positioning services across extreme environments is maintained. Temperature resilience is demonstrated with clock bias RMS confined to ≤0.19 ns and frequency stability preservation is ensured with Allan deviation fluctuations constrained within ≤15% under combined thermo-mechanical stresses.

Consequently, this proposed method provides a viable and efficient solution for advanced time–frequency reference in LEO navigation constellations, fundamentally transforming OCXO capabilities by concurrently delivering atomic-clock-grade stability and crystal-oscillator efficiency. 

Future work will focus on radiation-hardened implementations for complex space environments.

## Figures and Tables

**Table 1:** FDCI model parameter list.

Dimension	Symbol	Constraint Formulation	Physical Parameter
Quantization noise	D1	$\frac{{u_{min}}^{2}}{12}\leq {2\tau^{2}\left(K\sigma_{osc}(\tau )\right)}^{2}$ *^1^ $u_{min}\geq 0.1\times \sigma_{osc}\left(\tau \right)$	Minimum step for micro-step
Phase continuity	D2	$2\pi \cdot N{\cdot f}_{ref}\cdot u_{max}\leq 0.1\;rad/s$	Maximum step for frequency adjustment
Long-term stability	D3	${0.5\times \sigma_{y_{atomic}}\left(\tau \right)\leq \Sigma }_{22}{{\leq \tau \cdot \sigma }^{2}}_{spec}\left(\tau \right)$, at least τ=100 s *^2^	Allan deviation
Accuracy	D4	${\left(0.02\;ns\right)}^{2}\leq \Sigma_{11}=\frac{Q_{11}+\tau^{2}\Sigma_{22}}{1-\lambda^{2}}\leq {\left(0.15\;ns\right)}^{2}$ *^3^	Clock bias error (RMS)

*^1^ K is 0.2 for empirical scale factor; *^2^ $\sigma_{spec}\left(\tau \right)$ is less than 1 × 10^−12^; *^3^ $\lambda =max\left|eig(A-BL_{LQR})\right|<1$.

**Table 2:** Comparative Allan deviation measurements (note: improvement ratios > 1 indicate stability degradation, and improvement ratios < 1 indicate stability enhancement).

Average Time (s)	Allan Deviation (Rubidium Clock)	Allan Deviation (Free-Running)	Allan Deviation (LS Fitting)	Allan Deviation (Optimized LQG)	Improvement Ratio1 (LS/Free)	Improvement Ratio2 (Optimize LQG/Free)
1	1.72 × 10^−12^	5.15 × 10^−13^	6.51 × 10^−13^	5.76 × 10^−13^	1.2659	1.1195
2	/	5.70 × 10^−13^	6.94 × 10^−13^	6.33 × 10^−13^	1.2182	1.1100
4	/	6.75 × 10^−13^	9.14 × 10^−13^	7.09 × 10^−13^	1.3535	1.0504
10	9.86 × 10^−13^	7.35 × 10^−13^	1.05 × 10^−12^	8.08 × 10^−13^	1.4313	1.0993
20	/	7.85 × 10^−13^	1.48 × 10^−12^	8.61 × 10^−13^	1.8790	1.0968
40	/	8.54 × 10^−13^	1.21 × 10^−12^	9.68 × 10^−13^	1.4180	1.1335
100	6.13 × 10^−13^	1.15 × 10^−12^	9.02 × 10^−13^	9.38 × 10^−13^	0.7843	0.8157
200		1.62 × 10^−12^	7.01 × 10^−13^	7.82 × 10^−13^	0.4317	0.4815
400		2.41 × 10^−12^	4.31 × 10^−13^	5.10 × 10^−13^	0.1788	0.2116
1000	2.17 × 10^−13^	4.17 × 10^−12^	2.49 × 10^−13^	2.34 × 10^−13^	0.0597	0.0561
2000		7.45 × 10^−12^	1.43 × 10^−13^	1.35 × 10^−13^	0.0192	0.0181
4000		1.29 × 10^−11^	8.85 × 10^−14^	8.50 × 10^−14^	0.0069	0.0066
10,000	4.18 × 10^−14^	4.90 × 10^−11^	4.25 × 10^−14^	4.22 × 10^−14^	0.0009	0.0009

**Table 3:** Comparative performance metrics (note 1: ↑ indicates improvement with percentage and ↓ indicates degradation; note 2: frequency deviation and frequency stability—lower values indicate better performance).

Metric	Free-Running OCXO	LS Fitting	Optimized LQG	LQG vs. Free (LQG/Free)	LQG vs. LS (LQG/LS)
Clock Bias peak to peak (ns)	930	0.9	0.3	↑ 99.97%	↑ 66.67%
Clock Bias RMS (ns)	420	0.46	0.14	↑ 99.89%	↑ 69.56%
Frequency Deviation	9.15 × 10^−11^	8.94 × 10^−12^	2.5 × 10^−12^	↑ 97.27%	↑ 72.04%
Stability @10 s (ADEV)	7.35 × 10^−13^	1.05 × 10^−12^	8.08 × 10^−13^	↓ 9.93%	↑ 23.05%
Stability @100 s (ADEV)	1.15 × 10^−12^	9.02 × 10^−13^	9.38 × 10^−13^	↑ 81.57%	↓ 3.90%
Stability @1000 s (ADEV)	4.17 × 10^−12^	2.49 × 10^−13^	2.34 × 10^−13^	↑ 94.39%	↑ 6.02%
Stability @10,000 s (ADEV)	4.90 × 10^−11^	4.25 × 10^−14^	4.22 × 10^−14^	↑ 99.91%	↑ 0.71%

**Table 4:** Environmental test results of performance metrics (note 1: clock bias RMS and frequency deviation are absolute with percent change, frequency stability (Allan deviation) is measured over the different intervals with percent change; note 2: ↑ represents improvement with percentage and ↓ represents degradation with percentage).

Condition	Clock Bias RMS (ns)	Frequency Deviation	Frequency Stability (ADEV τ = 1 s)	Frequency Stability (ADEV τ = 10 s)	Frequency Stability (ADEV τ = 100 s)	Frequency Stability (ADEV τ = 1000 s)	Frequency Stability (ADEV τ = 10,000 s)
Baseline (25 °C)	0.14	4.3 × 10^−12^	6.25 × 10^−13^	7.68 × 10^−13^	9.2 × 10^−13^	2.2 × 10^−13^	4.41 × 10^−14^
+45 °C	0.19 (↓ 35%)	5.42 × 10^−12^ (↓ 26%)	5.72 × 10^−13^ (↑ 8%)	7.5 × 10^−13^ (↑ 2%)	1.03 × 10^−12^ (↓1 2%)	2.25 × 10^−13^ (↓ 2%)	4.42 × 10^−14^ (↓ 0.2%)
+10 °C	0.17 (↓ 21%)	5.48 × 10^−12^ (↓ 27%)	5.31 × 10^−13^ (↑ 15%)	8.61 × 10^−13^ (↓ 12%)	1.01 × 10^−12^ (↓ 9%)	2.51 × 10^−13^ (↓ 14%)	4.24 × 10^−14^ (↑ 4%)
0 °C	0.16 (↓ 14%)	5.52 × 10^−12^ (↓ 28%)	6.13 × 10^−13^ (↑ 2%)	7.64 × 10^−13^ (↑ 0.5%)	9.57 × 10^−13^ (↓ 4%)	2.22 × 10^−13^ (↓ 0.9%)	4.01 × 10^−14^ (↑ 9%)
−10 °C	0.16 (↓ 14%)	4.63 × 10^−12^ (↓ 7%)	5.73 × 10^−13^ (↑ 8%)	8.85 × 10^−13^ (↓ 15%)	8.99 × 10^−13^ (↑ 2%)	2.21 × 10^−13^ (↓ 0.45%)	4.13 × 10^−14^ (↑ 6%)
−20 °C	0.18 (↓ 28%)	5.36 × 10^−12^ (↓ 24%)	6.32 × 10^−13^ (↑ 1%)	7.4 × 10^−13^ (↑ 3.6%)	8.53 × 10^−13^ (↑ 7%)	2.4 × 10^−13^ (↓ 9%)	4.63 × 10^−14^ (↓ 5%)
Random Vibration	0.16 (↓ 12%)	4.67 × 10^−12^ (↓ 8.6%)	5.23 × 10^−13^ (↑ 16%)	7.99 × 10^−13^ (↓ 4%)	1.02 × 10^−12^ (↓ 10%)	2.45 × 10^−13^ (↓ 11%)	4.38 × 10^−14^ (↑ 0.6%)
Sinusoidal Vibration	0.17 (↓ 21%)	5.45 × 10^−12^ (↓ 26%)	5.31 × 10^−13^ (↑ 15%)	7.66 × 10^−13^ (↑ 0.3%)	9.25 × 10^−13^ (↓ 0.5%)	2.35 × 10^−13^ (↓ 7%)	4.39 × 10^−14^ (↑ 0.6%)

## Data Availability

Data are contained within the article.

## Abbreviations

The following abbreviations are used in this manuscript:

LEO	Low Earth Orbit
GNSS	Global Navigation Satellite System
OCXO	Oven-Controlled Crystal Oscillator
RT	Real Time
PPS	Pulse Per Second
LQR	Linear Quadratic Regulator
KF	Kalman Filter
LS	Least Squares
LQG	Linear Quadratic Gaussian
FDCI	Four Dimension Constraint Integrated
STS	Short-Term Stability
LTS	Long-Term Stability
